# Borderline Personality Disorder: Risk Factors and Early Detection

**DOI:** 10.3390/diagnostics11112142

**Published:** 2021-11-18

**Authors:** Paola Bozzatello, Claudia Garbarini, Paola Rocca, Silvio Bellino

**Affiliations:** Department of Neuroscience “Rita Levi Montalcini”, University of Turin, Via Cherasco 15, 10126 Turin, Italy; paola.bozzatello@unito.it (P.B.); claudia.garbarini@unito.it (C.G.); paola.rocca@unito.it (P.R.)

**Keywords:** borderline personality disorder, early onset, diagnosis, early detection, risk factors

## Abstract

Personality disorders (PDs) exert a great toll on health resources, and this is especially true for borderline personality disorder (BPD). As all PDs, BPD arises during adolescence or young adulthood. It is therefore important to detect the presence of this PD in its earlier stages in order to initiate appropriate treatment, thus ameliorating the prognosis of this condition. This review aims to highlight the issues associated with BPD diagnosis in order to promote its early detection and treatment. To do so, we conducted a search on PubMed database of current evidence regarding BPD early diagnosis, focusing on risk factors, which represent important conditions to assess during young patient evaluation, and on diagnostic tools that can help the clinician in the assessment process. Our findings show how several risk factors, both environmental and genetic/neurobiological, can contribute to the onset of BPD and help identify at-risk patients who need careful monitoring. They also highlight the importance of a careful clinical evaluation aided by psychometric tests. Overall, the evidence gathered confirms the complexity of BDP early detection and its crucial importance for the outcome of this condition.

## 1. Introduction

As stated in the Diagnostic and Statistical Manual of Mental Disorders (5th ed.), borderline personality disorder (BPD) is a serious mental illness characterized by a pervasive pattern of instability in interpersonal relationships, self-image and affect, as well as marked impulsivity. This condition arises in early adulthood or adolescence, leading to severe functional impairment and subjective discomfort [1].

The term borderline was first introduced in 1938 by Stern, who used it to describe a group of patients situated on the border between the neurotic and the psychotic group, associated with a peculiar resistance to psychotherapeutic treatment [2]. Later on, in 1979, Spitzer and colleagues formulated the original diagnostic criteria for BPD, which consisted of instability of affect, identity disturbance and deficiency of impulse control [3]. In the subsequent editions of the Diagnostic Manual of Mental Disorders, whose latest instalment is represented by DSM-5, these criteria were further expanded, including frantic efforts to avoid real or imagined abandonment, a pattern of unstable and intense interpersonal relationships, recurrent suicidal behavior, chronic feelings of emptiness, difficulty controlling anger, and transient, stress-related paranoid ideation or severe dissociative symptoms Box 1 [1].

Box 1 **DSM-5 diagnostic criteria for borderline personality disorder-modified.**

A pervasive pattern of instability in several areas (interpersonal relationships, self-image and affects) associated with marked impulsivity, which arises in adolescence or early adulthood and can be recognized in a variety of contexts, as indicated by five (or more) of the following criteria:Intense fear of abandonment, which subjects frantically try to avoid, be it real or imagined.A tendency to have unstable and intense interpersonal relationships, which alternate between extremes of idealization and devaluation.Identity disturbance, characterized by markedly and persistently unstable self-image or sense of self.Impulsivity in at least two potentially self-damaging contexts (e.g., spending, sex, substance use, reckless driving, binge eating).Recurrent suicidal (gestures or threats) or self-mutilating behaviorMarked reactivity of mood leading to affective instability (e.g, intense episodic dysphoria, irritability or anxiety, usually lasting a few hours and only rarely more than a few days).Chronic feelings of emptiness.Difficulty controlling anger, which is often inappropriate or excessive (e.g., frequent displays of temper, constant anger, recurrent physical fights).Transient and stress-related paranoid ideation or severe dissociative symptoms.Modified from DSM-5, APA, 2013

The median population prevalence of BPD is estimated to be around 1.6% but is probably much higher due to underdiagnosis. In primary care settings it can reach about 6%, increasing to 10% in outpatient mental health clinics and as high as 20% among psychiatric inpatients. There is evidence that this prevalence may decrease in older age groups [1].

As regards gender distribution, in the past decades, evidence seemed to suggest a greater prevalence of BPD among females, as is still stated in DSM-5, which reports a M:F ratio of 1:3 [1]. However, it was later observed that these differences appear to be due to diagnostic or sampling inaccuracies or to biological or sociocultural factors, as found in the NESARC study performed by Tomko and colleagues in 2014. In this study, only a minimal prevalence difference was observed between males and females (2.4% vs. 3.05%) [4].

BPD, as all personality disorders (PDs), does not suddenly arise during adulthood. In fact, prodromal signs and symptoms can already be observed in younger age, especially during adolescence [5,6,7,8]. Alas, studies that assess the prevalence of PDs in youths and adolescents are still scarce [9]. In the general population, cumulative prevalence rates of BPD in 16-year-olds and 22-year-olds are 1.4% and 3.2%, respectively, while in mental health settings, this disorder can be found in 11% of young psychiatric outpatients and in as many as 50% of inpatients [7,9,10,11].

BPD can present a variable outcome; most commonly, it causes chronic instability during early adulthood, resulting in episodes of serious affective and impulsive dyscontrol, which require demanding levels of health resources. In fact, the societal impact of PDs can be found in emotional suffering, disability and economic burden. In BPD especially, the suicide rate can be as high as 8–10% [12]. What is more, the presence of a PD also interferes with response to treatment of physical and psychiatric comorbidities, such as migraine, HIV, anxiety disorders and substance use disorders [13]. Furthermore, PDs, and BPD in particular, are associated with high rates of unemployment, absences from work and inefficiency at work, with only 25% of patients suffering from BPD working full time and 40% receiving disability payments [14].

Even though the impact on global functioning and the risk of self-harm and suicide generally decrease with advancing age, allowing subjects to obtain a greater stability in interpersonal relationships and vocational functioning in their 30s and 40s [1,15], BPD is associated with higher inpatient and outpatient mental health service use than other psychiatric disorders [16].

Compared with other PDs, children and adolescents suffering from BPD present increased rates of hospitalization due to suicidal ideation or attempts [17], more severe comorbid pathology [18] and poorer clinical and psychosocial functioning [19,20], similar to what is observed in adults. Indeed, evidence shows that early borderline pathology (before 19 years of age) represents a predictor of long-term impairment in functioning and of longer duration of symptoms—for as long as 20 years [21,22].

Unfortunately, diagnosis and treatment of BPD is often delayed, leading to a less favorable outcome. This is especially true in patients with early onset BPD, in whom the detection and subsequent therapeutic intervention on the disorder is usually further put off [23]. Indeed, in the last two decades, there has been a great sensibilization towards PD diagnosis in adolescence, reflected in the significant increase in empirical studies regarding this matter [24,25], and in the legitimization of adolescent PD diagnosis in psychiatric nomenclature (DSM-5 [1] and ICD-11 [26]), as well as in national treatment guidelines in the UK [27] and Australia [28]. Nonetheless, a recent review by Sharp has stressed how scientific evidence and national practice guidelines are yet to penetrate routine clinical care [29]. Indeed, in common clinical practice diagnosis—and consequently, treatment—of BPD is usually delayed due to underestimation of symptoms and, often, hesitation to diagnose this disorder in younger individuals [23,30]. Recent research has recognized the main reasons for this reluctance in five issues [7,29]: (1) the retained belief that psychiatric nomenclature does not allow the diagnosis of PD in adolescence; (2) certain features of PD are normative and not particularly symptomatic of personality disturbance (this is especially true when trying to distinguish between physiological adolescent upheaval and BPD symptoms [21]); (3) the symptoms of PD are better explained by other psychiatric syndromes [26,31]; (4) adolescents’ personalities are still developing and therefore too unstable to warrant a PD diagnosis; and (5) considering that PD is a long-lasting, treatment-resistant and unpopular-to-treat condition, it would be stigmatizing to label an adolescent with BPD [26,32].

In particular, BPD detection during adolescence is also impaired by the fact that this disorder shows different clinical features in teenagers and adults. In fact, early-onset BPD is more likely to present with the more executive symptoms of the disorder (e.g., recurrent self-harm and suicidal behavior, other impulsive and self-damaging behavior, and inappropriate anger), while enduring characteristic symptoms (e.g., unstable relationships and identity disturbances) are more often diagnosed among adults [33]. Thus, the clinical presentation of BPD is prone to variations over time, showing different individual symptoms during personality development. However, an accurate diagnosis can still be achieved by considering the core dysfunctional areas of this condition (e.g., affective dysregulation, interpersonal disturbance and impulsivity or behavioral dysregulation) [34]. This suggests that, given the low categorical stability of BPD in adolescence, a dimensional approach might better account for the developmental variability and heterogeneity observed during this age period, helping in the diagnosis process [30].

The reluctance to diagnose BPD can cause great damage to patients since early identification of this disorder represents the key to promoting early intervention programs, which should guarantee appropriate treatments in youths. In fact, some retrospective studies in adult subjects showed that the mean age for first psychiatric contact was 17–18 years and that inability to recognize BPD at first presentation resulted in an impossibility of setting up early interventions [35,36]. 

Given all that was stated above, it follows that conditions in childhood and adolescence that involve a high risk of progression to BPD should be carefully monitored [23]. Several factors were identified as predictors of early BPD onset: precocious environmental factors, child and adolescent temperamental characteristics, early psychopathological features and neurobiological correlates [23]. These factors should be carefully assessed when evaluating a young patient in order to identify a precocious phase of BPD and consequently plan an adequate follow-up and/or intervention [23,37,38]

To further aid in this assessment, several diagnostic tools have been designed in the last decades, of which psychometric and projective personality tests are still the more readily available. However, new evidence has been found regarding neuroimaging, instrumental and possibly even genetic assessment, even though on this latter aspect, findings are still quite inconsistent [7].

The aim of the present review is to provide clinicians with an up-to-date evaluation of the available evidence in the literature regarding early detection of BPD in order to aid in the diagnostic process. In particular, we focused on articles dealing with risk factors for BPD useful for recognizing at-risk individual, as well as clinical and instrumental diagnostic strategies for this disorder.

## 2. Materials and Methods

In May 2021, we conducted an electronic search on the database Medline about early diagnosis of BPD, BPD in adolescence and risk factors for BPD using the following search string: “borderline personality disorder” AND “early diagnosis” OR “borderline personality disorder” AND “adolescence” OR “borderline personality disorder” AND “risk factors”. This search led to a total of 684 studies. We further implemented the results of this search by repeating it using the following three strings with MeSH restrictions: (“Borderline Personality Disorder”[Mesh]) AND “Early Diagnosis”[Mesh] (12 results), (“Borderline Personality Disorder”[Mesh]) AND “Adolescent”[Mesh] (1884 results), and finally (“Borderline Personality Disorder”[Mesh]) AND “Risk Factors”[Mesh] (462 results). These strings ensured a highly sensitive search for the published works indexed in Medline. Overlapping studies were excluded. We included the following types of publications: controlled trials, observational studies, longitudinal and prospective studies, cohort studies, and reviews from January 2000 until May 2021 concerning early diagnosis of BPD in young age and adolescence as the main topic. A limitation of this review is that Medline was the only database used to search articles. What is more, we only included publications written in the English language.

## 3. Results

A total of 171 records were included in the present review. The selection was operated as follows: 770 works were excluded for dating before 1 January 2000, a further 178 records because of being written in a language other than English, while another 66 were not considered due to the unavailability of the full text. Eligibility status for the remaining articles was determined according to the following criteria: (1) all studies were screened on the basis of title and abstract; (2) papers that passed the initial screening were reviewed on the basis of a careful examination of the full manuscript content. Thus, this review included 171 records, including 33 reviews, 7 commentaries/expert articles, 61 longitudinal studies, 67 cross-sectional studies, 1 case-report study, and 2 controlled trials.

## 4. Discussion

### 4.1. Risk Factors

In the last decades, several risk factors involved in the onset of BPD have been identified in clinical research. The risk for BPD, in fact, arises from the interaction between genetic predisposition and adverse life experiences, which can be more frequently present in the history of these subjects due to temperamental factors [39]. Thus, vulnerability to BPD generally arises from precocious environmental factors, which are mainly related to family condition, early trauma and child psychopathological features — with associated neurobiological alterations — that can often progress into a clinically significant personality disorder during adolescence [23].

#### 4.1.1. Precocious Environmental Factors

A broad range of environmental factors were found to play a role in the onset of BPD, as shown in several studies investigating the development of this disorder, which can be found in Table 1 [8,40,41,42,43,44,45,46,47,48,49,50,51,52,53,54,55,56,57,58,59,60,61,62,63,64,65,66,67,68,69,70,71,72,73,74,75,76,77,78,79,80,81,82,83,84,85,86,87,88,89,90,91,92,93,94,95]. Among these at-risk conditions, the most relevant are represented by socioeconomic status, family psychopathology, parent-child relationship and traumatic events, such as child abuse, maltreatment or neglect. A special mention is required for the effect of social influences, like bullying and rejection by peers [23].

#### Family-Related Factors

As regards family-related risk factors, Cohen and colleagues found that lower socioeconomic background predicted BPD symptoms and that this effect was maintained stably over time [40]. The same conclusion was reached by Crawford and colleagues [41] and is confirmed by three studies by Stepp and colleagues, which showed that poverty requiring public assistance may predict BPD symptoms during adolescence [42,43].

Another important aspect to consider is represented by parent-child relationship. A fairly recent meta-synthesis of the systematic reviews written on the influence of parenting on the development of personality disorders was performed in 2019 by Steele and colleagues, who systematically analyzed eight systematic reviews. Five of these reviews found that maladaptive parenting — such as low warmth, rejection, low maternal satisfaction with child, hostility and harsh discipline/punishment, disrupted maternal communication, maternal-expressed negative emotion and maternal inconsistency and over-involvement — represented a psychosocial risk factor for the development of borderline personality pathology [8,44,45,46,47]. Three works found that borderline personality pathology was associated with maladaptive parenting, negative offspring and parenting-offspring outcomes [48,49,50]. Steele and colleagues thus recommend that a greater emphasis should be put on parenting in clinical practice, with the development of parenting interventions for individuals with personality disorders [51].

Concerning clinical studies that researched this topic, a follow-up study by Stepp and colleagues found that mother-child discord predicted BPD in adulthood (30 years) [52]. Moreover, as observed in a naturalistic study evaluating the effect of inadequate parent-child boundaries performed by Vanwoerden and colleagues (2017), relationships centered on guilt induction, psychological control and triangulation appear to be linked to BPD features in adolescents with severe behavioral and emotional disorders [53]. This is also supported by the finding made by Lyons-Ruth and colleagues that role confusions and disoriented behaviors in parent-child relationship are present in patients with early BPD symptoms, such as self-injury and suicidality in late adolescence [54]. Infurna and colleagues (2016) observed how BPD patients reported lower care from both mother and father, as well as higher levels of over-control and intrusiveness from both parents. At the same time, both mothers and fathers of BPD patients themselves reported inadequate levels of affection, warmth, empathy, and closeness from their parents, especially from their mother; in fact, univariate regression showed that a low level of motherly care reported by both mothers and fathers of the patients was significantly associated with BPD outcomes in offspring [55]. However, these results were not always consistent; in two studies, no significant correlation was observed between parenting style and the early onset of BPD [43,56].

It is interesting to note that this correlation between inadequate parent-child relationship and early BPD symptoms might also be influenced by genetic predisposition (see “Genetic and neurobiological factors”). For instance, Hammen and collaborators (2015) found a significant association between poor parent-child relationship and BPD onset at 20 years in subjects who presented a particular genotype for the oxytocin receptor gene (AA/AG), which conferred increased vulnerability [58].

As could be expected, the combined presence of family adversities and maladaptive parental behavior also determines an increased vulnerability for the development of personality disorders, as supported by Winsper and colleagues, who found that both these conditions were associated with increased risk for BPD in pre-adolescence (11 years of age) [59].

Strictly correlated with the importance of parent-child relationship is the impact of parents’ psychopathology on the development of BPD in the offspring. This is especially true for maternal BPD, which has been referred to as a putative precursor for BPD in children and adolescents [60,61], as supported by several studies. Three longitudinal studies, respectively performed by Reinelt, Stepp and Barnow and colleagues [52,62,63], consistently found that maternal BPD represented a predictor of BPD onset in adolescence (15 years) [62,63] and early adulthood (24 years) [52]. Mahan and colleagues also found that maternal psychological control was associated with borderline features in mothers and affective instability in offspring with an increased risk of developing BPD [64]. It is interesting to note that, as found by Steele and colleagues, the strength of BPD features in parents appears to be directly proportional to the level of stress perceived and to the impairment of competence in their parenting role and. Furthermore, the strength of BPD features in parents also appears to be associated with insecure attachment styles and poorer reflective capacity [65]. Therefore, helping to improve personality and mental health functioning of parents suffering from BPD, thus increasing parental reflective capacity and strengthening parent-child attachment relationships, may reduce parenting stress and increase parenting competence in these individuals, with a consequential positive effect on offspring as well.

The impact not only of borderline traits but also of other maternal psychopathological dimensions was evaluated in clinical research. Conway and colleagues observed that maternal externalizing disorders (conduct disorder, oppositional defiant disorder, ADHD, impulsive/aggressive behavior, self-injuries and substance use disorder) were associated with offspring internalizing disorders (depression and anxiety disorders, dissociative symptoms and suicidality) and BPD onset [66]. Winsper and colleagues found that maternal anxiety and depression during pregnancy are predictors for early BPD in offspring [22]. Stepp and colleagues also suggest a possible correlation, though not confirmed in the final analyses of their longitudinal study, between depressive symptoms and antisocial personality disorder in caregivers and the onset of BPD during adolescence [43]. On the contrary, there appears to be no correlation between maternal ego integration and impulsivity, medical problems and interpersonal disturbances and the onset of BPD symptoms during childhood/adolescence, according to several studies [41,56].

Paternal psychopathology, although less researched, was also found to play a significant role: Infurna and colleagues found that high levels of paternal depression and psychosocial stress were significantly associated with BPD in offspring [55].

#### Trauma-Related Factors

The correlation between early traumatic events and BPD has been widely researched in the last decades, receiving an increasing amount of scientific validation, albeit not always consistent. In fact, it has been observed that early traumas represent a trigger for the development of several BPD traits, such as affect instability, emotional dysregulation and self-destructive behaviors (i.e., substance abuse and self-harm conducts) [68]. These traumatic experiences are mainly represented by verbal, emotional, physical and sexual abuse, emotional and physical neglect and chronic exposure to peer victimization (bullying) [23,69].

Bozzatello and collaborators (2020) evaluated several traumatic factors using both a qualitative (ACE-IQ) and a quantitative criterion (CTQ-SF) in a sample of BPD outpatients. These factors were then correlated with the assessment of symptoms of BPD (impulsivity, socio-occupational functioning and subjective quality of life) by multiple regression analysis, finding that the role of trauma depends more on its presence than on its intensity and that the effects on BPD onset are approximately the same when patients experience an active behavior of abuse or are subjected to conditions of neglect [69].

In a longitudinal study conducted by Rogosch and colleagues on six-year-olds, it was observed that maltreatment was associated with lower agreeableness, conscientiousness and openness to experience, as well as higher neuroticism, with less adaptive personality clusters, especially in children subjected to both abuse and neglect. These characteristics were found to be stably maintained at nine years of age [70].

Indeed, Geselowitz and collaborators observed that adverse childhood experiences (ACEs), involving psychological and physical trauma, parental mental illness and poor socioeconomic background occurring in preschool age, represented the stronger predictor for BPD symptoms in adolescents (14–19 years old), even when parental psychopathology and poverty were excluded from the analysis [71].

Evidence is quite consistent in identifying sexual abuse as an important predisposing factor for early BPD onset, as supported by several studies [42,71,72,73,74,75,76,77,78] Moreover, it appears that adolescents with BPD present a higher risk of being involved in sexual trauma compared not only to healthy controls, as suggests the preliminary evidence collected by Venta and collaborators [74] but also to patients suffering from other psychiatric disorders. This finding is supported by Horesh and colleagues, who observed that in these subjects, there is a higher rate of sexual-abuse-related events than in depressed or in healthy adolescents [78]. What is more, there appears to be quite a specific correlation between childhood sexual abuse (CSA) and BPD, as supported by Rajan and collaborators, who observed that BPD was the only diagnosis absent in adolescents prior to the first registered CSA episode and that it started to be detected in the first year afterwards, then drastically increasing during the second year [77]. This finding is further corroborated by Infurna and colleagues, who found that sexual abuse was the only type of abuse to be independently correlated to early BPD onset in a group of female adolescents [75]. This association is also particularly relevant on a clinical level because it can alter clinical manifestations and worsen the course of BPD. In particular, it increases the risk of suicidal conducts [76,79], it has been associated with psychotic-like experiences [80], with a generally more severe symptomatology, especially in subjects with a history of prolonged CSA [71,81], and a lower remission rate [42,72]. Only one study we evaluated, conducted by Hecht and colleagues on middle-childhood subjects, was not consistent with this evidence, finding no correlation between sexual abuse and BPD, probably due to small sample size and the young age of the participants (symptoms usually become evident at a later age) [82].

Physical abuse has been widely researched as a predisposing condition for the development of PDs. Maltreatment and inherited vulnerability, in fact, play a synergic role to foster borderline personality traits. Belsky and collaborators observed that children who were physically abused presented a higher score of BPD symptoms at age 12 and were especially vulnerable if they had a family history of psychiatric disorders [83]. What is more, it was also found that a low level of the temperamental dimension of affiliation was associated with an earlier onset and a higher severity of BPD symptoms in children subjected to physical abuse [84]. The association between physical trauma and PDs, especially BPD, can be explained by considering that physical maltreatment negatively affects several personality domains, leading to affective instability, identity problems, negative relationships and self-harm. In fact, Hecht and collaborators observed that physically abused children scored higher on each of the aforementioned personality domains and presented more severe borderline traits and a higher risk for development of BPD compared to controls [82].

As regards verbal abuse, alas, as of today, data are still limited. Nonetheless, the available evidence suggests that harsh speech, as well as physical abuse, can have a traumatic impact. In fact, Johnson and colleagues found that children subjected to maternal verbal abuse were more than three times as likely compared to those who were not to develop borderline, narcissistic, obsessive-compulsive and paranoid PD during adolescence or early adulthood [85].

A much larger amount of research has been performed concerning the correlation between neglect, both physical and emotional, and early BPD onset. Indeed, higher rates of neglect were found in adolescents with BPD and concomitant depression, compared to healthy controls, by Goodman and colleagues [73]; the same was observed for neglect from both parental figures by Infurna and collaborators [75]. Moreover, physical neglect was found to be associated with earlier onset of BPD features by Hecht and collaborators [82]. In this regard, it has been suggested by Jovev and colleagues that the combination of specific temperamental traits and neglect (physical or emotional) may accelerate the presentation of BPD and antisocial personality disorder symptoms [84]. Two specific kinds of neglect were found to be associated with BPD (and also other cluster B PDs in the case of the first one): childhood supervision neglect (failure to set limits, to attend to misbehavior and to know child’s whereabouts and friends), as observed by Johnson and collaborators [86], and maternal withdrawal in infancy, as found by Lyons-Ruth and colleagues [87].

Finally, in more recent times, greater attention has been bestowed upon the role of social-group interactions, especially peer relationships, in the onset of psychopathology [23]. Victimization in the context of bullying during childhood, in fact, can have a negative impact on internal working models pertaining to relationships, impairing the ability to appropriately trust and interact with other people, thus leading to unstable relationships, distorted perception and emotional dysregulation and hence to a greater risk for BPD [88] Wolke and collaborators observed that any type of peer victimization during primary school represented a potential predictor for BPD symptoms at 12 years of age; a greater risk (up to sevenfold compared to controls) was conferred by exposure to combined (overt and relational) or chronic bullying [89]. Lereya and colleagues, moreover, found that being subjected to bullying between 7 and 10 years of age increased the risk of self-injury during late adolescence, especially if combined with an adverse family environment [90]. Even more so, Winsper and collaborators observed that peer victimization represented a significant predictor for BPD, depressive and psychotic symptoms in children with dysregulated behavior [91], which was confirmed by Haltigan and Vaillancourt on a sample of children with a reactive temperament [92]. Interestingly, Antila and colleagues found that only female, and not male, victims of bullying presented a fourfold increased risk for PDs, including BPD [93].

However, evidence is not always consistent in determining a strict causal correlation between trauma and BPD onset, or at least not when abuse is the sole factor considered. Firstly, in a study performed by Lyons-Ruth on adolescents, it was observed that the effect of maternal withdrawal was independent of and additive to the effect attributable to severity of childhood abuse so that both these conditions had to be present to determine a significant risk for BPD [87]. Furthermore, Bornalova and colleagues suggested that the association between childhood abuse and BPD traits arises from common genetic predisposition that can also overlap with internalizing and externalizing disorders so that BPD traits in adulthood appear to be better accounted for by heritable vulnerabilities [94]. Other authors suggest that genetic predisposition plays a modulating role in this context. For instance, Cicchetti and collaborators observed that oxytocin receptor (OXTR) and FK506 binding protein 5 (FKBP5) polymorphisms played a role in the sensibilization to the effect of maltreatment in the development of BPD traits, with a differential pattern between genders: in females, the gene-environment interaction was more consistent with the diathesis-stress model, and an increased risk for borderline symptoms was associated with the presence of minor alleles of the two candidate genes; in males, on the other hand, a differential-sensitivity interaction effect was observed, and a greater vulnerability was associated with major alleles [95].

#### 4.1.2. Temperamental and Personality Factors

Several temperamental characteristics and personality traits, commonly referred to as “intrapsychic factors”, have been found to predispose to BPD onset. Studies concerning this topic are listed in Table 2. It is important to assess for these features during clinical evaluation to ensure an early detection of this disorder [23]. Among these predisposing characteristics found in both children and adolescents can be enlisted affective instability, negative emotionality, inappropriate anger, poor emotional control, impulsivity and aggression [37,83,96,97,98] Less researched, on the contrary, is the correlation between childhood personality traits and BPD in adulthood [56,94,99]

Several studies investigated the correlation between temperament or personality features and early BPD symptoms. Joyce and colleagues observed that elevated harm avoidance and novelty seeking, associated with adverse childhood experiences and adolescent psychopathology, represented predictors for early BPD onset [100]. Similarly, Kaess and collaborators found that high harm avoidance, high novelty seeking and low reward dependence predispose to the development of this disorder [105].

Another feature found to be associated with BPD onset is aggression during childhood and adolescence. Cramer and collaborators observed that aggression and impulsivity in 11-years-olds predict the presence of BPD features at 23 years of age [110]. Interestingly, Crick et al. and Underwood observed that relational aggression but not physical aggression acted as a predictor for BPD traits [96,104]. Moreover, Vaillancourt and colleagues found that different types of aggression predicted the diagnosis of BPD at age 14 according to gender: relational aggression in males, physical aggression in females [98].

Negative emotionality—and more specifically, negative affectivity and poor emotional control—was also found to predict BPD onset. Lenzenweger and colleagues observed that negative emotionality and low constraint predicted BPD at 19 years, while lower agency predicted worsening of BPD traits over time [60]. Other authors found a correlation between the association of negative affectivity and impulsivity in childhood and BPD onset [8,37,43,103,108]. In fact, low self-control, impulsivity and affective instability are three tightly connected dimensions, all of which represent predictors for BPD when observed in adolescence, as supported by several studies [83,101,102].

Finally, another temperamental trait that was less addressed in research is anger. Crawford and colleagues, in fact, found a significant correlation between anger/tantrum dimension in childhood and BPD symptoms [41].

When evaluating the impact of these temperamental characteristics on the onset of BPD, it is important to consider their interaction with environmental and neurobiological factors. Indeed, as observed by Jovev and collaborators, it appears that low emotional control interacts with childhood maltreatment in predisposing to BPD traits during early and middle adolescence and that parental abuse can exert a moderating role if associated with low affiliation [84]. Similarly, Sharp et al. and Stepp et al., respectively, reported that the impact of low self-control and negative affectivity in promoting early BPD onset was modulated by harsh familial discipline and family adversities [42,109]. Martìn-Blanco and colleagues, moreover, found that the dimensions of neuroticism-anxiety and aggression-hostility, as well as emotional abuse, represented independent risk factors associated with BPD [106]. From a neurobiological standpoint, this is also supported by the finding made by Jovev and collaborators that vulnerability to BPD due to temperamental factors is also associated with alterations in hippocampal structure, more precisely with rightward hippocampal asymmetry [107].

#### 4.1.3. Genetic and Neurobiological Factors

Although BPD has been found to present a significant heritability, data available on this matter are still scarce and often inconsistent [111]. The current estimate is of heritability around 0.70, as observed in several studies conducted on twins [112,113,114]. Studies researching this matter are shown in Table 3.

**Figure 1:**
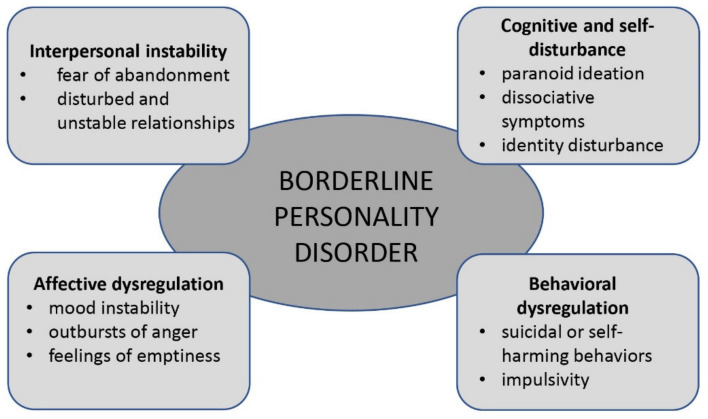
Adapted from Gunderson et al. 2018.

BPD and its four symptom phenotypes (interpersonal instability, cognitive and/or self-disturbance, affective and/or emotional dysregulation, behavioral dysregulation) (Figure 1) have been observed to aggregate in families [115,116,117], and a study performed on twins by Gunderson and colleagues found that a common family environment seemed to play little part in this aggregation, suggesting the presence of genetic predisposition [119]. In a genome-wide association study performed on a large number of patients with BPD by Witt and colleagues, a genetic overlap with other major psychiatric disorders was observed (bipolar disorder, schizophrenia and major depressive disorder). It is interesting to note that the implicated genes include *DPYD* (dihydropyrimidine dehydrogenase), *PKP4* (plakiphilin 4) and *SERINC5* (serine incorporator 5), which play an important role in basic properties of neural processing, such as cell adhesion and myelination [124]. Thus, genetic predisposition to BPD is likely not specific for this disorder, and this might imply the possibility that genetic overlap is linked to transdiagnostic clinical symptoms instead of reflecting an increased risk for psychiatric disorders in general [111]. However, a review conducted by Amad and collaborators did not detect a significant association between BPD and typical candidate genes for vulnerability for psychiatric disorders, such as *SLC6A4* (serotonin transporter) [127].

Other genetic polymorphisms that were observed in patients suffering with BPD, but also in other psychiatric disorders, affect genes involved in hypothalamic-pituitary-adrenal (HPA) axis activity, such as *FKBP5* and *CRHR*, which were found to be present, especially in patients exposed to childhood maltreatment [123,128,129]. Indeed, abnormalities in HPA axis hormones, the most important of which is excessive cortisol secretion, might mediate the negative effect of early-life adversities on the developing brain structure and function, especially on the affect-regulation circuitry [111]. In particular, this might imply functional and structural changes in the hippocampus [130] and amygdala [125], as well as the latter’s connections with the anterior cingulate cortex (ACC) [120]. Moreover, a higher biological synchrony was observed between parent and child HPA axis activity when they were in contact with one another, reflecting the poor quality of the parent-child interaction [126]. Another hormone that could be involved in the context of parent-child interaction is oxytocin, the levels of which increase in both counterparts when a fine-tune behavioral synchrony is present [118].

Not only genetic polymorphisms, but also epigenetic alterations, probably resulting from early-life maltreatment, are likely to play an important role in the development of BPD symptoms [111]. Amongst the genes that were found to be increasingly methylated in this disorder are *MIR124-3*, the product of which is involved in regulation of neural plasticity and amygdala function [122], and *BDNF* (brain-derived neurotrophic factor) [121].

Unfortunately, genetic and neurobiological factors do not present sufficient specificity for early detection or treatment, as happens with all psychiatric disorders [111].

### 4.2. Diagnosis of BPD

Studies dealing with the diagnosis of BPD are shown in Table 4.

#### 4.2.1. Diagnostic Criteria: DSM-5 and ICD-10

The DSM-5 recognizes nine diagnostic criteria for BPD [1] [Box 1], which, according to Gunderson and colleagues, can be grouped into four phenotypes (interpersonal instability, cognitive and/or self-disturbance, affective and/or emotional dysregulation, behavioral dysregulation) (Figure 1), consistent with the general criteria for a PD [111]. In order to make a BPD diagnosis, at least five of the nine criteria must be present [1]. Interestingly, Zimmerman and collaborators observed that meeting increasing numbers of these criteria is associated with greater illness severity only at the subthreshold level, i.e., up to five criteria [132]. It is important to underline that, as with other psychiatric disorders in DSM-5, the BPD diagnostic criteria define an independent category, which overlaps with other disorders. In this regard, it has been observed that the presence of even only one BPD criterion is useful to recognize, among patients suffering from other mental illnesses, those at greater risk for current or past suicidal ideation, history of psychiatric hospitalization or higher functional impairment [131]. Despite the fact that all criteria for BPD are weighed equally in DSM-5, two studies found that the “unstable relationships” criterion shows the best combined sensitivity and specificity for BPD [166] and presents the strongest familiar aggregation [119]. On the other hand, the “chronic feelings of emptiness” criterion was found to be the most strongly associated with psychosocial morbidity (including history of suicide attempts), hospitalization, global functional impairment and comorbidity with other mental disorders [133].

In the ICD, Tenth Revision (ICD-10), BPD is referred to as “emotionally unstable personality disorder”, characterized by instability in three areas: sense of self, relationships with other people and emotions [167]. With the forthcoming ICD, Eleventh Revision (ICD-11), scheduled to come into use in January 2022, great change is on the way in the field of personality-disorder assessment. In this latest classification system, in fact, the authors decided to apply a dimensional approach to these disorders, replacing all but one of the ICD-10 categorical personality disorder syndromes with a five-domain dimensional-trait model. These five domains will be represented by negative affectivity, detachment, dissociality, disinhibition, and anankastia (or obsessionality). The clinician will first assess the patient with respect to a level of personality-disorder severity based on the degree of self and/or interpersonal dysfunction (ranging from sub-threshold personality difficulty through to mild, moderate or severe personality disorder), and then can choose to also rate the person with respect to the five trait domains, along with a borderline pattern qualifier. The borderline label was thus the only one to have been retained due to concerns expressed by researchers and clinicians alike with respect to the great amount of evidence collected over the years on this disorder, especially regarding its treatment [26,168].

#### 4.2.2. Clinical Assessment

As supported by several studies, internalizing (i.e., depression, anxiety, dissociative symptoms, suicidality) [52,66,83,109,134,135,139,140,169] and externalizing psychopathology (i.e., conduct disorder, oppositional-defiant disorder, ADHD, impulsive-aggressive behavior, self-injuries and substance use disorder) [18,43,52,83,134,135,137,138,140,141,169] can often be observed in adolescents before BPD onset, with a possible predominance of internalizing symptoms in females and of externalizing features in males. Moreover, other clinical entities, such as obsessive-compulsive disorder (OCD), separation anxiety disorder and social phobia, can also frequently be present [7,18,19,30] According to Wolke and colleagues, indeed, any Axis I diagnosis may predict very early BPD onset (at age 12) [89]. It has been suggested that these conditions represent a predisposing background on which, under the influence of biological vulnerability and stressful life events, personality psychopathology develops during adolescence. Thus, it is very important to promptly detect these precocious stages of psychological suffering in order to prevent the onset of serious personality disorders, such as BPD [136,170].

From the considerations stated above stems the fact that patients suffering from BPD often come to clinical attention in the midst of an episode of another mental disorder, such as major depressive disorder (MDD), anxiety disorders, trauma-related disorders or substance use disorder (SUD). In some instances, the reason for seeking medical help might be a current crisis, interpersonal (often a relationship break-up) or of another nature (for example, job loss, school failure or a major life transition). In the most severe cases, the subject could present after a suicide attempt or other impulsive and self-destructive actions [111].

Assessment of patients with suspected BPD most commonly starts with a clinical interview. Since personality is defined by the way people see, relate to and think about themselves, others and the environment, this poses unique challenges because the perception of one’s own personality cannot be objective, as it is influenced by personality itself. Therefore, instead of directly questioning subjects about their personality, clinicians usually look for patterns in the way patients describe themselves, their interpersonal relationships and their work functioning. Moreover, important clues are provided to clinicians by the way patients interact with them during the interview and, very importantly, by information acquired from people close to the patient, such as parents, siblings or spouses. Considering the nature of the disorder, it is important to expect emotional intensity, anger, neediness, demanding behavior and tendencies to either overvalue or devalue the clinician. Great care should be bestowed upon differential diagnosis and comorbidity assessment [111].

#### 4.2.3. Structured Diagnostic Interviews and Self-Report Questionnaires

Clinical assessment of patients with suspect BPD can be exposed to several biases, such as interpretation, information and criterion variance (i.e., respectively, overgeneralization of present experience with the clinician to other life situations, reliance on general information not sufficient to evaluate specific BPD criteria and deviation from individual criteria incurring in BPD underdiagnosis or overdiagnosis without a basis) [171]. Therefore, in order to contrast these sources of diagnostic unreliability, in common clinical practice, the use of semi-structured and fully structured diagnostic interviews and self-report questionnaires specific for the diagnosis of BPD and other PDs is encouraged [111]. In fact, available evidence shows how these tools prove to be more reliable and valid than routine clinical assessments for the diagnosis of these disorders so that the combined use of interview and self-report represents the optimal strategy to correctly identify BPD [142,143].

As regards semi-structured interviews, they most commonly include questions to gather information to determine whether or not a subject meets each of the diagnostic criteria, applying diagnostic algorithms for all DSM-5 PDs. They primarily differ from one another in the arrangement of these questions, either by type of disorder or by area of functioning (Table 5). All semi-structured interviews are designed to be administered by a trained and experienced clinician [111]. Amongst these interviews, the most frequently used is the Structured Clinical Interview for DSM-5 Personality Disorders (SCID-5). Moreover, some interviews are specific for the evaluation of BPD, such as the Borderline Personality Disorder Severity Index (BPDSI) and the Zanarini Rating Scale for BPD (ZAN-BPD). This latter scale is a nine-item, validated, clinician-based diagnostic interview that assesses the severity of DSM-IV-based borderline personality disorder symptoms (subdivided into four categories: affective, cognitive, impulsive and interpersonal) and also measures meaningful changes in symptoms over time.

Concerning self-report questionnaires, these instruments present the advantage of greatly expediting diagnostic assessments and are particularly useful in the context of first-stage screening evaluations [111]. They differ from one another in their structure, length and specificity for BPD (Table 6). It is interesting to note that due to their characteristics, these questionnaires can be administered to the general population for screening purposes, and in this context, the affective instability criterion has proven to be the most sensitive and specific for BPD diagnosis [144]. What is more, a recent study performed by Fung et al. in China found promising results concerning online assessment of BPD using a web-based version of the Self Report-Dissociative Disorders Interview-Borderline Personality Disorder (SR-DDIS-BPD) [145]. Other self-report questionnaires, moreover, are available to evaluate degree of personality functioning [111].

Regarding BPD diagnosis in adolescents, some of the semi-structured interviews and self-report questionnaires developed for adults have proven to also be effective in younger subjects, such as the BPD items of the Structured Clinical Interview for DSM-IV Axis II Personality Disorders Personality Questionnaire (SCIDII- PQ), the Borderline Personality Questionnaire (BPQ), the McLean Screening Instrument for BPD (MSI-BPD) and the Personality Assessment Inventory-Borderline Scale (PAI-A-BOR). However, other tools were specifically developed for children and adolescents. Three semi-structured interviews have been validated in under-18 teenagers: the Childhood Interview for Borderline Personality Disorder (CIBPD), the Revised Diagnostic interview for Borderlines (DIB-R) and the BPD Severity Index IV Adolescent Version (BPDSI-IV-Adolescent). The Shedler-Westen Assessment Procedure for Adolescents, Version II, BPD scale (SWAP-II-A-BPD) was designed for use by clinically experienced observers in the context of either a thorough examination of a patient using a systemic clinical research interview or in a professional clinical assessment. Among self-report questionnaires, of note are the Borderline Personality Features Scale for Children (BPFSC), its short-form version (BPFSC-11) and the parent-report version of the BPFSC (BPFS-P) [7,172].

#### 4.2.4. Laboratory and Instrumental Assessment

As for other psychiatric disorders, no specific laboratory or instrumental tests are available for the diagnosis of BPD. Nevertheless, it is recommended to subject the patient to routine blood tests in order to rule out other medical conditions, such as thyroid imbalance, as well as a neuroimaging assessment through computer tomography (CT) scan—or preferably, magnetic resonance (MR) scan—to exclude the possibility of the presence of organic lesions.

Concerning neuroimaging studies, interestingly, several unspecific structural changes have been observed in young subjects suffering with BPD in whom the burden of confounders (such as prolonged duration of illness, pharmacotherapy and recurring trauma) is considered to be less pronounced [146,148]. These structural anomalies mainly affect grey and white matter in fronto-limbic areas, which are deeply involved in emotion regulation and impulse control [23]. More specifically, in a study by Chanen et al., a right-sided loss of orbitofrontal cortex (OFC) grey matter was observed in BPD patients [146], while in a study conducted by Brunner et al., a significant shrinking of both left OFC and bilateral dorsolateral prefrontal cortex (DLPFC) was found in comparison with a group of healthy subjects, although no differences were found comparing BPD patients with subjects suffering from other mental disorders [148]. In the same sample of BPD patients, Maier-Hein et al. observed lower myelination and less organized white-matter bundles in bilateral fornices, accompanied by white matter-disrupted connections in the thalamus, hippocampus and heteromodal association cortex, suggesting that adolescents with BPD present a dysfunctional emotional-processing network [152]. Richter et al. further observed reduced dimension of the right amygdala compared to healthy (but not clinical) controls and smaller hippocampal volume compared to both healthy and clinical controls [153]. Walterfang et al., on the other hand, found no difference in size of the corpus callosum compared to healthy controls [149]. In two different studies, both Whittle et al. and Goodman et al. observed a volume reduction in the anterior cingulate cortex (ACC) in adolescents with BPD [73,147]. In a diffusion tensor imaging (DTI) study, New et al. found decreased fractional anisotropy in three fiber bundles in BPD subjects compared to controls: inferior longitudinal fasciculus (connecting the temporal and occipital lobes) and uncinate fasciculus and occipitofrontal fasciculus (connecting parts of the limbic system to the OFC and other frontal regions) [151]. Finally, regarding hippocampal alterations, in an already-quoted study by Jovev and colleagues, a rightward hippocampal asymmetry was observed in BPD pre-adolescents (11–13 years old), which was associated with temperamental alterations, such as high affiliation in males and low effortful control in females [107].

In addition to the morphological alterations mentioned above, some functional anomalies in the brains of subjects suffering from BPD, albeit aspecific, have been appreciated using fMRI scans. For example, a hyperactivation in the medial prefrontal cortex, temporal parietal junction, several regions of the frontal pole, the precuneus and middle temporal gyrus has been detected by Beeney et al.; interestingly, this was associated with scarce temporal consistency in self and other representations and poor differentiation between self and others [154]. What is more, Doering and collaborators observed that a significantly decreased level of deactivation in the anterior and posterior cortical midline structures was able to predict low levels of personality functioning and identity integration [150]. In this regard, autobiographical memory—a process of reflective thinking through which links are formed between elements of life and self—can be considered as an indirect index of identity integration. This function was found to be altered in subjects with BPD by Bozzatello and colleagues in the context of both resolved and unresolved life events and was associated with hyperactivity in several brain areas, such as the right anterior cingulate cortex (ACC), the right medial prefrontal cortex (MPFC), and the right dorsolateral prefrontal cortex (DLPFC) —for resolved life events—, bilateral ACC, bilateral DLPFC, and right temporo-parietal junction—for unresolved life events—, ACC and DLPFC—for both conditions [155].

As regards electroencephalogram (EEG), no specific alterations were found in the literature. In a study by Pop-Jordanova and colleagues, lower frequency and coherence were observed exclusively in low bands (delta and theta) in BPD patients compared to a healthy group, with dubious significance [156]. Another study, performed by Arikan and colleagues, compared the EEG recordings of three groups—BPD patients, bipolar patients and healthy subjects—finding significant differences only between healthy controls and psychiatric patients but no differences between the two clinical groups, suggesting the need for further research [157].

#### 4.2.5. Differential Diagnosis and Comorbidities

As stated above, BPD subjects often present psychiatric comorbidities, which need to be carefully assessed [160,173]. Amongst the mental illnesses that can be found in association to BPD figure mood disorders (major depressive disorder (MDD) and bipolar disorder), anxiety disorders, stressor-related disorders (acute stress disorder and PTSD), substance-related disorders, dissociative disorders, disrupted-behavior disorders, somatoform disorders, neurodevelopmental disorders (such as ADHD) and other personality disorders. The type and frequency of psychiatric comorbidities varies according to population assessed (i.e., clinical or general population), clinical setting (inpatient, outpatient or sub-specialty clinic), prevalence of the disorders in the population, duration of the disorders and methods of assessment [111]. The most commonly found comorbidities are represented by MDD and anxiety disorders, with a median lifetime prevalence, respectively, of 71% and 88% [111]. It has been observed that BPD complicates the treatment of other mental disorders and is associated with a greater tendency to maintain a chronic course [161,162].

As regards PTSD, the nature of the association between this condition and BPD is, indeed, quite controversial, considering the numerous similarities shared by these disorders, such as clinical features (particularly major disturbances in emotional and affect regulation, impulse control, reality testing, interpersonal relationships, self-evaluation and sense of identity), the psychopathological importance of trauma, a possible common genetic vulnerability and the presence of similar neurofunctional abnormalities [174]. It is interesting to note that the entity known as complex PTSD, originally described as a syndrome related to precocious exposure to multiple traumatic experiences and characterized by the presence of difficulties in emotion regulation, interpersonal relationships and self-concept, has recently been linked to BPD. In fact, these disorders share some psychopathological features, such as dissociative symptoms, dysregulation of emotions, self and relational disturbances [175]. Chronic early exposure to particular types of traumatic experiences often results in more pervasive disorders than simple PTSD, involving impairment of attachment style and of the ability to modulate emotions. Trauma can indeed produce its effects on behavioral, emotional, physiologic and neuroanatomical levels, and later stressors tend to be experienced by victims of trauma as a reactivation of the precocious traumatic experiences. It has been suggested that emotional dysregulation and impulsivity in subjects suffering from BPD may increase vulnerability to repetitive exposure to traumatic events due to high appraisal of threat, diminished coping resources, increased exposure to risky situations and intense emotional responding peri-traumatically. Exposure to multiple traumatization, at the same time, negatively affects the sense of self and emotion-regulation strategies, and these key alterations of personality can evolve into BPD or cPTSD [158]. What is more, emotional dysregulation may also increase the tendency to perceive new events as threatening and traumatic [176].

Regarding other psychiatric comorbidities, in a similar way, these are unlikely to be distinct and independent entities from BPD [111]. For instance, in the case of depression, often, depressed BPD patients do not satisfactorily respond to antidepressants, and depressive symptoms can remit with improvement of BPD [159,165]. The same can be stated for anxiety disorders [164]. Moreover, the diagnosis of multiple disorders to describe a patient’s symptoms is encouraged by the tendency of the DSM to split up psychopathology [111].

Distinguishing BPD from other mental disorders is probably less important than setting priorities for treatment, considering how effective intervention in this disorder proves to be beneficial in diminishing associated psychopathology [163,164].

## 5. Conclusions

Considering all that was stated above, it is evident that BPD represents a relevant issue, with its clinical and socioeconomic repercussions, already at a young age. In order to deal with this condition, early detection appears to be of crucial importance. To do so, screening for the presence of the most common risk factors in youths represents the first step towards the monitoring of at-risk individuals and the establishment of a specific treatment. Among these risk factors, available evidence shows how precocious environmental factors (family-related and trauma-related) interact with temperamental and personality factors—associated with genetic and neurobiological correlates—in the pathogenesis of this personality disorder.

As seen in the last part of this review, the diagnosis of BPD remains essentially based on clinical assessment, implemented with structured diagnostic interviews and self-report questionnaires. Laboratory and instrumental assessment can prove to be useful tools to rule out the presence of medical comorbidities, the symptomatology of which can be mistaken for a psychiatric disorder like BPD. Finally, differential diagnosis with other psychiatric conditions, like mood disorders, must always be considered.

## Figures and Tables

**Table 1:** Studies on precocious environmental factors.

Family-related factors				
Study	Study Design	Sample	Trial Duration	Outcomes
Cohen et al., 2008 [40]	Longitudinal study; community population	A random residence-based cohort of children and young adults between ages 10 and 36	26 years	Low family socioeconomic status → STPD and BPD in offspring
Crawford et al., 2009 [41]	Longitudinal study; community population	766 youths aged 13 to 33	21 years	Extended maternal separations before age 5 → offspring BPD symptoms
Carlson et al., 2009 [56]	Longitudinal study; community population	A sample of young mothers and their first-born children (N = 162; 82 males, 80 females)	28 years	BPD symptoms in adulthood related to endogenous and environmental history in early childhood
Winsper et al., 2012 [59]	Cohort study; community population	6050 mothers and their children (age range 10.4–13.6 years, mean age 11.7 years)	11 years	Family adversity → BPD symptoms of offspring
Stepp et al., 2013 [52]	Longitudinal cohort study; community population	1709 students 14–18 years old (360 with a history of a depressive disorder and 284 with a history of mood disorders) vs. 457 HC	16 years	Maternal-child discord, maternal BPD, paternal SUD + proband depression, SUD and suicidality associated with later BPD symptoms
Reinelt et al., 2013 [62]	Longitudinal cohort study; community population	295 children (15–20 years old) and their biological mothers drawn from the population-based Greifswald Family Study	5 years	Maladaptive mother-child interactions → longitudinal transmission of borderline symptoms from mother to child
Barnow et al., 2013 [63]	Longitudinal cohort study; community population	323 offspring (15–20 years old) and their mothers from the community-based Greifswald Family Study	5 years	Maternal BPD symptoms and depression → BPD and general psychopathology in offspring
Stepp, Whalen et al., 2014 [43]	Longitudinal cohort study; community population	2451 girls aged 14–17 drawn from the Pittsburgh Girls Study	3 years	Only-child characteristics, and not caregiver psychopathology, predicted BPD symptoms
Stepp et al., 2015 [37]	Longitudinal study; community population	113 at-risk adolescent girls aged 16–18	3 years	Family adversity → ↑ BPD symptoms during late adolescence in offspring
Lyons-Ruth et al., 2015 [54]	Cross-sectional study; community population	120 young adults	-	BPD traits → more role for confusion and more disoriented behavior in the interaction with the parent
Hammen et al., 2015 [58]	Longitudinal study; community population	385 youths (235 females, 150 males, offspring of mothers with a probable history of depression)	20 years	OXTR rs53576 moderates the link between early family quality and later BPD symptoms
Conway et al., 2015 [66]	Longitudinal study; community population	700 at-risk youths (15–20 years old)	5 years	Maternal externalizing disorder history, offspring internalizing disorder history, family stressors and school-related stressors → ↑ BPD risk
Winsper et al., 2015 [22]	Longitudinal study; community population	6050 mothers and their children (11–12-year-olds)	12 years	Prenatal anxiety and depression → BPD in offspring
Infurna et al., 2016 [55]	Cross-sectional study; clinical inpatient population	91 female adolescent psychiatric inpatients (M_age_ = 15.57 years), along with 87 mothers and 59 fathers	-	Low maternal care and paternal psychiatric symptoms → adolescent BPD in offspring
Vanwoerden et al., 2017 [53]	Cross-sectional study; clinical inpatient population	301 adolescent (65.1% female; ages 12–17) inpatients	-	Parental guilt induction and psychological control → children’s BPD features
Mahan et al., 2018 [64]	Cross-sectional study; clinical outpatients and community population	28 mothers with a diagnosis of BPD and 28 HC with male and female adolescents aged 14–18	-	Mothers with BPD use more total psychological control with their children → adolescent affective instability
Steele et al., 2020 [65]	Cross-sectional study; community population	284 parents (94.72% female, *M*_age_ = 37.37), of which 69 (24.30%) met BPD criteria	-	Individuals high in BPD features → ↑ stress and ↓ competence in their parenting role
**Trauma-related factors**				
**Study**	**Study Design**	**Sample**	**Trial Duration**	**Outcomes**
Johnson et al., 2000 [86]	Longitudinal study; community population	738 youths and their mothers	18 years	Childhood emotional, physical and supervision neglect → ↑ risk for PDs + ↑ PD symptom levels during adolescence and early adulthood
Johnson et al., 2001 [85]	Longitudinal study; community population	793 mothers and their offspring assessed in four waves (at ages 5, 14, 16 and 22 years)	18 years	Maternal verbal abuse during childhood → BPD, NPD, OCPD and PPD during adolescence or early adulthood
Rogosch et al., 2004 [70]	Longitudinal study; community population	211 six-year-old children (135 maltreated and 76 non-maltreated)	3 years	Six-year-old maltreated children → ↓ agreeableness, conscientiousness and openness to experience + ↑ neuroticism, maintained at age 9
Horesh et al., 2008 [78]	Cross-sectional study; community and clinical population	19 adolescents with MDD, 20 with BPD and 20 matched controls	-	The BPD group reported more sexual abuse LE than the control and MDD groups
Horesh et al., 2009 [79]	Retrospective study; community and clinical population	40 suicidal adolescents and young adults with MDD (22) or BPD (18), 40 non-suicidal adolescents and young adults with MDD (20) and BPD (20), 40 HC	-	Suicidal BPD participants reported more lifetime sex-abuse-related LE compared with non-suicidal BPD participants
Biskin et al., 2011 [71]	Longitudinal study; community and clinical population	47 adolescent girls (31 with BPD, 16 HC)	10 years	Unremitting BPD associated with a current episode of MDD, lifetime SUD and self-reported childhood sexual abuse
Staebler et al., 2011 [88]	Cross-sectional study; community and clinical population	35 patients with BPD and 33 HC	-	A negative bias for perceived social participation and ambiguous facial emotional expressions → disturbed relatedness in patients with BPD
Venta et al., 2012 [74]	Cross-sectional study; clinical inpatient population	147 adolescent BPD inpatients	-	Adolescents with BPD more likely to have a history of sexual trauma and to report sexual concerns
Belsky et al., 2012 [83]	Longitudinal cohort study; community population	1116 pairs of same-sex twins	12 years	Exposure to harsh treatment in the family environment through age 10 → BPD-related characteristics at age 12
Wolke et al., 2012 [89]	Longitudinal study; community population	6050 mothers and their children drawn from the Avon Longitudinal Study of Parents and Children (ALSPAC)	11 years	Victims of peer bullying and of chronic victimization → ↑ risk of BPD symptoms
Jovev et al., 2013 [84]	Longitudinal study; community population	245 children (aged 11–13)	2 years	Childhood neglect → ↑ BPD symptoms; childhood abuse → ↑ ASPD symptoms
Lyons-Ruth et al., 2013 [87]	Longitudinal study; community population	Adolescents (M_age_ 19.9 years) from 56 families participating in a longitudinal study since infancy	18 years	Maternal withdrawal in infancy → BPD symptoms + suicidality/self-injury in late adolescence
Lereya et al., 2013 [90]	Longitudinal study; community population	4810 children and adolescents drawn from the Avon Longitudinal Study of Parents and Children (ALSPAC) cohort assessed for bullying exposure (7–10 years old) and self-harm (16–17 years old)	10 years	Being bullied → ↑ risk of self-harm, directly and indirectly, via depression symptoms in early adolescence
Bornovalova et al., 2013 [94]	Longitudinal study; community population (twins)	Over 1300 pairs of twins (11 to 24 years old)	13 years	Common genetic influences that also overlap with internalizing and externalizing disorders → association between childhood abuse and BPD traits
Hecht et al., 2014 [82]	Cross-sectional study; community population	314 maltreated and 285 non-maltreated children (M_age_ = 11.30) from comparable low socioeconomic backgrounds	-	Maltreated children had more severe BPD features, according to chronicity, patterns of onset and recency of maltreatment
Cicchetti et al., 2014 [95]	Cross-sectional study; community population	1051 maltreated and non-maltreated low-income children	-	Different pattern of gene-environment interaction according to gender
Stepp et al., 2015 [42]	Longitudinal study; community population	113 at-risk adolescent girls aged 16–18	3 years	Exposure to adversity → ↑association between negative emotional reactivity and BPD symptoms
Infurna et al., 2016 [75]	Cross-sectional study; clinical population	44 female adolescent inpatients with BPD and 47 CC with mixed psychiatric diagnoses	-	Sexual abuse, general family functioning and low maternal care → adolescent BPD
Kaplan et al., 2016 [76]	Longitudinal study; clinical population	58 female youths with BPD aged 13–21 years with (*n* = 29) and without (*n* = 29) a history of child abuse	2 months	Child abuse (particularly co-occurring physical and sexual abuse) → ↑ risk for NSSI + suicidality among BPD youths.
Haltigan et al., 2016 [92]	Longitudinal study; community population	566 Canadian children assessed yearly from ages 8 to 16	8 years	Parent- and child-reported mental health symptoms + peer relations risk factors + intra-individual risk factors: significant predictors of personality psychopathology
Winsper et al., 2017 [91]	Retrospective study; community population	7159 children drawn from the Avon Longitudinal Study of Parents and Children (ALSPAC), assessed from birth to 14 years	14 years	Bully victimization associated with BPD symptoms
Antila et al., 2017 [93]	Longitudinal study; clinical inpatient population	508 adolescent inpatients (300 girls, 208 boys)	12 years	Female victims of bullying (but not boys) → ↑ likelihood of developing a PD later in life, especially BPD
Sengutta et al., 2019 [80]	Cross-sectional study; clinical inpatient population	200 inpatients aged 16–21 years with non-psychotic disorders	-	Childhood trauma (emotional neglect and sexual abuse) → psychotic-like experiences, with the mediation of BPD features
Turniansky et al., 2019 [81]	Retrospective study; clinical inpatient population	78 female adolescent inpatients with BPD with (*n* = 38) and without (*n* = 40) a history of prolonged childhood sexual abuse (CSA)	8 years	Prolonged CSA → ↑ duration and ↑ number of psychiatric hospitalizations + ↑ rate of NSSI and suicidal attempts, smoking, alcohol use and sexual impulsivity.
Bozzatello et al., 2020 [69]	Cross-sectional study; clinical outpatient population	68 BPD outpatients	-	Earlier onset of BPD mainly associated with traumatic events (abuse, neglect, dysfunction in household environment and bullying)
Rajan et al., 2020 [77]	Longitudinal cohort study; community population	519 girls aged 12–17 with registration of CSA experience in their medical record and 4920 age-matched HC	7 years	CSA → ↑ risk for suicide attempts, stress disorders, psychosis and alcohol abuse + ↑ healthcare consumption patterns and drug prescriptions
Geselowitz et al., 2021 [71]	Longitudinal cohort study; community population	170 children drawn from a prospective longitudinal study of early childhood depression, assessed at ages 3, 6, 14 and 19	16 years	Preschool ACEs, internalizing symptoms and low maternal support → BPD symptoms + preschool and school-age suicidality

BPD, borderline personality disorder; ASPD, antisocial personality disorder; DSM, Diagnostic and Statistical Manual of Mental Disorders.

**Table 2:** Studies on temperamental and personality factors.

Study	Study Design	Sample	Trial Duration	Outcomes
Joyce et al., 2003 [100]	Cross-sectional study; clinical outpatient population	180 depressed outpatients	-	Childhood abuse and/or neglect + borderline temperament + childhood and adolescent depression, hypomania, conduct disorder and alcohol and drug dependence → BPD
Crick et al., 2005 [96]	Longitudinal study; community population	400 (54% female) fourth through sixth graders	1 year	BPD features moderately stable over the course of the study, with girls reporting higher levels of BPD features than boys
Carlson et al., 2009 [56]	Longitudinal study; community population	A sample of young mothers and their first -born children (N = 162; 82 males, 80 females)	28 years	Endogenous and environmental history in early childhood → disturbance of child functioning in middle childhood/early adolescence → BPD symptoms in adulthood
Tragesser et al., 2009 [101]	Cross-sectional study; community population	141 undergraduates	-	Both affective instability and impulsivity uniquely associated with BPD features
Gratz et al., 2009 [102]	Cross-sectional study; community population	263 children aged 9 to 13	-	Effect of affective dysfunction and disinhibition in childhood BPD symptoms mediated by self- and emotion-regulation deficits
Tragesser et al., 2010 [103]	Longitudinal study; community population	353 young adults (aged 18–20)	2 years	Negative affect predictive of most BPD symptoms but not future impulsive behavior
Underwood et al., 2011 [104]	Longitudinal study; community population	255 children aged 9 to 13 (131 girls and 124 boys)	5 years	High levels of social and physical aggression in middle childhood → greatest risk for adolescent psychopathology (BPD and NPD)
Belsky et al., 2012 [83]	Longitudinal cohort study; community population	1,116 pairs of same-sex twins followed from birth through age 12 years	12 years	BPD-related characteristics more common in children who had exhibited poor cognitive function, impulsivity and more behavioral and emotional problems at age 5 years
Bornalova et al., 2013 [94]	Longitudinal study; community population (twins)	A large sample of twins (over 1300 pairs) aged 11–24	13 years	Common genetic influences that also overlap with internalizing and externalizing disorders → association between childhood abuse and BPD traits
Kaess et al., 2013 [105]	Cross-sectional study; community and clinical population	33 adolescents with BPD, 35 clinical controls (CCs) and 31 healthy controls (HCs), all females	-	↑ novelty seeking + ↑ harm avoidance + ↓ reward dependence in the adolescents with BPD
Stepp, Keenan et al., 2014 [37]	Longitudinal cohort study; community population	2450 girls aged 5–8 at first evaluation, 14–19 at second evaluation, drawn from the Pittsburgh Girls Study	5 years	Childhood temperament dimensions of emotionality, activity, low sociability and shyness predict adolescent BPD symptom development
Nelson et al., 2014 [97]	Longitudinal cohort study; community population	168 preschool children (84 boys, 84 girls) living in intact, two-parent biological households	10 years	Preschool relational aggression + aversive parenting → ↑ aggression + BPD features in adolescent females; preschool authoritative parenting → ↓ aggression and BPD features in boys, vs. authoritarian parenting → ↑ aggression
Vaillancourt et al., 2014 [98]	Longitudinal cohort study; community population	484 youths (aged 10 to 14)	4 years	Childhood relational aggression + depression for boys; physical and relational aggression + depression + ADHD for girls → BPD features at age 14
Martín-Blanco et al., 2014 [106]	Cross-sectional study; clinical population	130 BPD subjects	-	Temperamental traits + childhood emotional abuse → development + severity of BPD
Jovev et al., 2014 [107]	Cross-sectional study; community population	153 healthy adolescents (M_age_ = 12.6 years)	-	Boys: ↑/↓ affiliation + hippocampal asymmetry → ↑ BPD symptoms; girls: ↓ effortful control + hippocampal asymmetry → ↑ BPD symptoms
Hallquist et al., 2015 [108]	Longitudinal study; community population	A sample of girls (aged 5 to 17) taken from the Pittsburgh Girls Study (*n* = 2450)	12 years	Harsh punishment + poor self-control + negative emotionality → BPD symptom severity at age 14; ↓ self-control ages 12–14 → ↑BPD symptoms from 14 to 17
Sharp et al., 2015 [109]	Longitudinal study; community population	730 adolescents	1 year	Experiential avoidance → BPD features + severity of BPD symptoms at 1-year follow-up
Cramer et al., 2016 [110]	Longitudinal study; community population	100 youths (aged 11 to 23)	12 years	Childhood personality traits (impulsivity + nonconformity/aggression) → adult BPD features
Conway et al., 2017 [99]	Longitudinal study; community population	2450 high-risk adolescent girls aged 14 to 20	6 years	BPD pathology fluctuates in response to situational influences

BPD, borderline personality disorder; ND, narcissistic personality disorder; HC, healthy controls; CC, clinical controls.

**Table 3:** Studies on genetic and neurobiological factors.

Study	Study Design	Sample	Trial Duration	Outcomes
Torgersen et al., 2000 [112]	Cross-sectional study; community population	92 monozygotic and 129 dizygotic twin pairs	-	PDs more strongly influenced by genetic effects than almost any axis I disorder and more than most broad personality dimensions (BPD heritability of 0.69)
Torgersen et al., 2008 [115]	Cross-sectional study; community population	1386 Norwegian twin pairs between the age of 19 and 35 years	-	Heritability of PD traits: ASPD 38%, HPD 31%, NPD 24%, BPD 35%
Kendler et al., 2008 [116]	Cross-sectional study; community population	2794 young-adult members of the Norwegian Institute of Public Health Twin Panel	-	Genetic risk factors → broad vulnerability to PD pathology and/or negative emotionality; environmental experiences → tendency of cluster A, B and C PDs to co-occur.
Bornovalova et al., 2009 [117]	Longitudinal study; community population	A large sample of adolescent female twins (aged 14–24) taking part in the Minnesota Twin Family Study (MTFS)	10 years	Both the stability and change of BPD traits are highly influenced by genetic factors and modestly by nonshared environmental factors.
Feldman et al., 2010 [118]	Cross-sectional study; community population	112 parents (71 mothers and 41 fathers) and their 4–6-month-old infants	-	Mothers with high levels of affectionate contact → ↑oxytocin following mother–infant interaction; fathers with high levels of stimulatory contact → ↑ oxytocin
Gunderson et al., 2011 [119]	Cross-sectional study; community and clinical population	A total of 368 probands (132 with BPD, 134 without BPD and 102 with MDD) and 885 siblings and parents of probands	-	Substantial familial aggregation of BPD
Torgersen et al., 2012 [113]	Cross-sectional study; community population	2,794 twins from the Norwegian Institute of Public Health Twin Panel	-	Heritability of Cluster B PDs: 0.30 if assessed by interview, 0.40-0.50 if assessed by self-report questionnaire (0.67 for BPD)
Veer et al., 2012 [120]	Cross-sectional study; community population	20 healthy male participants	-	Endogenous cortisol levels may modulate amygdala functional connectivity with specific regions in the medial PFC, even under relatively stress-free circumstances
Perroud et al., 2013 [121]	Cross-sectional study; community and clinical population	115 subjects with borderline personality disorder (BPD) and 52 controls	-	BPD subjects → ↑ methylation status in BDNF gene; after I-DBT ↓ methylation status in responders (→ changes in depression, hopelessness and impulsivity scores), ↑ in non-responders
Reichborn-Kjennerud et al., 2015 [114]	Longitudinal study; community population	2282 Norwegian twins in early adulthood	10 years	Genetic risk factors → ASPD and BPD trait stability from early to middle adulthood; transient environmental risk factors → phenotypic change.
Prados et al., 2015 [122]	Cross-sectional study; clinical population	96 BPD subjects suffering from a high level of child adversity and 93 subjects suffering from MDD and reporting a low rate of child maltreatment	-	Several genes differently methylated, either in BPD compared with MDD or in relation to the severity of childhood maltreatment
Martín-Blanco et al., 2016 [123]	Cross-sectional study; community and clinical population	481 subjects with BPD and 442 controls	-	Several HPA axis genetic variants in BPD subjects with sexual and physical abuse
Witt et al., 2017 [124]	Cross-sectional study; community and clinical population	998 BPD patients and 1545 controls	-	BPD overlaps with BD, MDD and schizophrenia on the genetic level
Iorio et al., 2017 [125]	Cross-sectional study; community population	308 college-attending, non-Hispanic European-Americans who completed the Duke Neurogenetics Study	-	Polygenic variation linked to HPA axis function → risk for anxiety symptomatology
Pratt et al., 2017 [126]	Cross-sectional study; community and clinical population	97 mothers (28 with MDD, 69 HC) with their 6-year-old children	-	Higher adrenocortical synchrony between mother and child → ↑ physiological stress and < adaptive dyadic relational patterns

BPD, borderline personality disorder; PDs, personality disorders; ASPD, antisocial personality disorder; ND, narcissistic personality disorder; HPD, histrionic personality disorder; MDD, major depressive disorder; HC, healthy controls; CC, clinical controls; PFC, pre-frontal cortex; BDNF, brain-derived neurotrophic factor; I-DBT, intensive dialectical behavior therapy; HPA, hypothalamic-pituitary-adrenal.

**Table 4:** Studies on BPD diagnosis.

Diagnostic Criteria				
Study	Study Design	Sample	Trial Duration	Outcomes
Zimmerman et al., 2012 [131]	Cross-sectional study; clinical population	3,200 psychiatric outpatients (1,976 with 0 or 1 DSM-IV criterion for BPD)	-	Low-severity levels of borderline personality disorder pathology can be determined reliably and have validity
Zimmerman et al., 2013 [132]	Cross-sectional study; clinical population	3,069 psychiatric outpatients	-	Dimensional scoring of BPD more important for subthreshold levels of pathology, less critical once a patient meets the diagnostic threshold
Ellison et al., 2016 [133]	Cross-sectional study; clinical population	1,870 adult psychiatric outpatients	-	BPD criteria of impulsivity, affective instability, emptiness and anger → dysfunction; emptiness → marker of impairment on all indices of psychosocial morbidity
**Clinical assessment**				
**Study**	**Study design**	**Sample**	**Trial duration**	**Outcomes**
Ramklint et al., 2003 [134]	Cross-sectional study; clinical population	158 former inpatients (*M*_age_ = 30.5 years)	-	Identification + treatment of childhood psychiatric disorders → ↓ risk for development of an adult PD
Thatcher et al., 2005 [135]	Longitudinal study; community and clinical population	355 adolescents with AUD and 169 adolescents without AUD aged 16 to 22 years	6 years	AUD and other adolescent psychopathology can culminate in BPD symptomatology
Chanen et al., 2007 [136]	Cross-sectional study; clinical population	177 psychiatric outpatients aged 15 to 18 years (46 with BPD, 88 with other PDs and 43 with no PDs)	-	BPD → psychopathology, general functioning, peer relationships, self-care and family and relationship functioning (> than Axis I disorders and other PDs)
Miller et al., 2008 [137]	Longitudinal study; community and clinical population	96 adolescents with ADHD and 85 HCs, aged 16 to 26 years old	3 years	Childhood ADHD → ↑ risk for PDs in late adolescence.
Belsky et al., 2012 [83]	Longitudinal cohort study; community population	1,116 pairs of same-sex twins followed from birth through age 12 years	12 years	BPD-related characteristics at age 12 years co-occurred with symptoms of conduct disorder, depression, anxiety and psychosis
Wolke et al., 2012 [89]	Longitudinal study; community population	6,050 mothers and their children drawn from the Avon Longitudinal Study of Parents and Children (ALSPAC)	11 years	Peer bullying and chronic victimization → ↑risk of BPD symptoms
Stepp et al., 2012 [138]	Longitudinal cohort study; community population	1,233 girls drawn from the Pittsburgh Girls Study, aged 10 to 14 years	4 years	↑ levels of ADHD and ODD scores at age 8 → BPD symptoms at age 14
Stepp et al., 2013 [52]	Longitudinal cohort study; community population	1,709 students (14–18 years old), of which 360 had a history of a depressive disorder and 284 had a history of mood disorders, compared with 457 HC	16 years	Maternal-child discord, maternal BPD, paternal SUD, + proband depression, SUD, and suicidality → later BPD symptoms
Ha et al., 2014 [20]	Cross-sectional study; clinical population	335 adolescent inpatients (aged 12–17 years), 33% with BPD	-	Adolescent inpatients with BPD → ↑rates of psychiatric comorbidity
Stepp, Whalen et al., 2014 [43]	Longitudinal cohort study; community population	2,451 girls aged 14 -17 drawn from the Pittsburgh Girls Study	3 years	Child impulsivity and negative affectivity, as well as caregiver psychopathology, were related to parenting trajectories, while only child characteristics predicted BPD trajectories.
Conway et al., 2015 [66]	Longitudinal study; community population	700 at-risk youths (15–20 years old)	5 years	Maternal externalizing disorders + offspring internalizing disorders + family stressors + school-related stressors → BPD risk
Sharp et al., 2015 [109]	Longitudinal study; community population	730 adolescents	1 year	Experiential avoidance → BPD features + levels of borderline symptoms at 1-year follow-up
Krabbendam et al., 2015 [139]	Longitudinal study; community population	229 detained adolescent females (mean age = 15.5 years)	6 years	Post-traumatic stress, depressive symptoms and dissociation during detention → ↑ risk for BPD in adulthood
Bo et al., 2017 [140]	Cross-sectional study; clinical population	109 adolescent patients with consecutive referrals to psychiatric clinic (45 with BPD, 64 CCs)	-	BPD group → ↓ mentalizing abilities + ↑ problematic attachments to parents and peers + ↑ self-reported levels of psychopathology
Koenig et al., 2017 [141]	Cross-sectional study; clinical and community population	77 adolescent psychiatric inpatients and 50 detainees	-	Lifetime self-injury behavior among adolescent psychiatric inpatients and detainees is associated with similar patterns of psychopathology and BPD
Bornovalova et al., 2018 [137]	Longitudinal study; community population	1,763 female twins aged 14 to 24	10 years	↑ levels of BPD traits → earlier onset and faster escalation of AUD and DUD → ↓ normative decline in BPD traits
**Structured diagnostic interviews and self-report questionnaires**				
**Study**	**Study design**	**Sample**	**Trial duration**	**Outcomes**
Zanarini et al., 2000 [142]	Cross-sectional study; clinical population	12 master’s or doctoral-level raters	-	Axis II disorders can be diagnosed reliably when using appropriate semi-structured interviews
Samuel et al., 2013 [143]	Longitudinal study; clinical population	320 patients in the Collaborative Longitudinal Personality Disorders Study diagnosed with PDs by therapist, self-report and semi-structured interview at baseline	5 years	Self-report questionnaire and semi-structured interview PD diagnoses → > predictive validity vs. PD diagnoses assigned by a treating clinician
Morey et al., 2016 [136]	Cross-sectional study; clinical population	337 clinicians and their target patients	-	Clinical diagnoses of PDs diverge from the rules designated in the DSM
Zimmerman et al., 2017 [144]	Cross-sectional study; clinical population	3674 psychiatric outpatients	-	Affective instability criterion: sensitivity of 92.8% (> than other 8 BPD criteria) + negative predictive value of 99%
Fung et al., 2020 [145]	Cross-sectional study; clinical population	828 subjects with web-based diagnosis of BPD	-	The web-based BPD measure could discriminate between participants with and without BPD
**Laboratory and instrumental assessment**				
**Study**	**Study design**	**Sample**	**Trial duration**	**Outcomes**
Chanen et al., 2008 [146]	Cross-sectional study; clinical and community population	20 BPD patients and 20 HCs	-	BPD patients → right-sided OFC grey-matter loss; no significant differences in amygdala or hippocampal volumes
Whittle et al., 2009 [147]	Cross-sectional study; clinical and community population	15 female BPD patients and 15 female HCs	-	↓ volume of the left ACC in BPD patients → parasuicidal behavior, impulsivity and fear of abandonment
Brunner et al., 2010 [148]	Cross-sectional study; clinical and community population	60 female right-handed individuals (aged 14–18 years): 20 with BPD, 20 CCs and 20 HCs	-	Early morphological changes in BPD are located in the PFC: reduced gray matter in the DLPFC bilaterally and in the left orbitofrontal cortex OFC
Walterfang et al., 2010 [149]	Cross-sectional study; clinical and community population	20 teenaged first-presentation BPD patients and 20 HCs	-	Gross neuroanatomical changes in the callosum are not present in teenagers with first-presentation BPD
Goodman et al., 2011 [73]	Cross-sectional study; clinical and community population	13 adolescent inpatients with co-morbid BPD and MDD and 13 HCs	-	↓ BA24 volume → ↑ number of suicide attempts and BPD symptom severity but not depressive symptoms
Doering et al., 2012 [150]	Cross-sectional study; clinical and community population	17 female BPD patients and 17 female HCs	-	Deactivation in the anterior and posterior cortical midline structures → ↓ personality functioning + ↓ identity integration
New et al., 2013 [151]	Cross-sectional study; clinical and community population	38 BPD patients (14 adolescents, 24 adults) and 32 HCs (13 adolescents, 19 adults)	-	In early onset BPD, the normal developmental “peak” in fractional anisotropy in ILF is not achieved → possible neural substrate for the OFC-amygdala disconnection in adults with BPD
Maier-Hein et al., 2014 [152]	Cross-sectional study; clinical and community population	20 adolescent patients with BPD (aged 14–18 years), 20 HCs, and 20 CCs	-	In BPD, white-matter alterations in pathways involved in emotion regulation + parts of the heteromodal association cortex related to emotion recognition
Jovev et al., 2014 [107]	Cross-sectional study; community population	153 healthy adolescents (M_age_ = 12.6 years)	-	Boys: ↑/↓ affiliation + hippocampal asymmetry → ↑ BPD symptoms; girls: ↓ effortful control + hippocampal asymmetry → ↑ BPD symptoms.
Richter et al., 2014 [153]	Cross-sectional study; clinical and community population	60 right-handed female adolescents between 14 and 18 years of age (20 patients with BPD, 20 CCs and 20 HCs)	-	In BPD, differences in the right and left hippocampus and in the right amygdala + ↓volume in frontal and parietal regions
Beeney et al., 2016 [154]	Cross-sectional study; clinical and community population	8 right-handed females (17 with BPD, 21 HCs) aged 18–60.	-	In BPD, ↓ maintenance of self and other representations + ↑ activation in medial PFC, temporal parietal junction, regions of the frontal pole, precuneus and middle temporal gyrus (areas crucial for social cognition)
Bozzatello et al., 2019 [155]	Cross-sectional study; clinical and community population	24 BPD patients and 24 HCs	-	Inefficient attempt to reconstruct a coherent narrative of life events → hyperactivity in ACC and DLPFC in BPD patients
Pop-Jordanova et al., 2019 [156]	Cross-sectional study; clinical and community population	10 BPD patients (5 males and 5 females, mean age 20.4 years), 10 HCs (6 males and 4 females, mean age 24.2 years).	-	EEG characteristics in BPD without statistical differences, except in low bands (delta and theta) → lower frequencies and coherence
Arikan et al., 2019 [157]	Cross-sectional study; clinical and community population	111 subjects (11 HCs, 25 BPD, 75 BD)	-	No significant differences in EEG characteristics between the two clinical groups
**Differential diagnosis and comorbidities**				
**Study**	**Study design**	**Sample**	**Trial duration**	**Outcomes**
Orbach et al., 2003 [158]	Cross-sectional study; clinical and community population	32 suicidal inpatients (14 males, 18 females), 29 non-suicidal inpatients (11 males, 18 females), aged 25–60; 98 HCs (75 females and 23 males), aged 19–39	-	Intense mental pain related to loss of life meaning and suicide
Gunderson et al., 2004 [159]	Longitudinal study; clinical population	161 BPD patients, with and without co-occurring MDD	3 years	Rate of remissions of BPD → not affected by co-occurring MDD; rate of MDD remissions → ↓ by co-occurring BPD
Eaton et al., 2011 [160]	Cross-sectional study; community population	34,653 civilian, non-institutionalized individuals aged ≥18 years	-	Complex patterns of co-morbidity in BPD → connections to other disorders (latent internalizing and externalizing dimensions)
Hasin et al., 2011 [161]	Longitudinal study; clinical population	1172 subjects with alcohol dependence, 454 with cannabis use disorder and 4017 with nicotine dependence, drawn from the National Epidemiologic Survey on Alcohol and Related Conditions (NESARC)	3 years	ASPD, BPD and STPD associated with persistent alcohol, cannabis and nicotine use disorders
Skodol et al., 2011 [162]	Longitudinal study; community population	1,996 participants in a national survey	3 years	APD, HPD, PPD, SPD, STPD and especially BPD → ↑ risk for MDD persistence
Gunderson et al., 2014 [163]	Longitudinal study; clinical population	223 BPD patients with co-occurring MDD (*n* = 161), bipolar I disorder (*n* = 34) and bipolar II disorder (*n* = 28)	10 years	BPD and MDD strongly related: ↑ time to remission and ↑ time to relapse; BPD and BD largely independent disorders, except BD II → ↑ BPD’s time to remission
Keuroghlian et al., 2015 [164]	Longitudinal study; clinical population	164 BPD patients with co-occurring GAD (*n* = 42), panic disorder with agoraphobia (*n* = 39), panic disorder without agoraphobia (*n* = 36), social phobia (*n* = 48), OCD (*n* = 36) and PTSD (*n* = 88)	10 years	BPD negatively affects the course of GAD, social phobia and PTSD; anxiety disorders have little effect on BPD course
Boritz et al., 2016 [165]	Randomized controlled trial	180 BPD patients	3 years	BPD + PTSD → ↑ levels of global psychological distress

BPD, borderline personality disorder, PDs, personality disorders, ASPD, antisocial personality disorder, NPD, narcissistic personality disorder, HPD, histrionic personality disorder, PPD, paranoid personality disorder, STPD, schizotypal personality disorder, SPD, schizoid personality disorder, APD, avoidant personality disorder, MDD, major depressive disorder, BD, bipolar disorder, SUD, substance use disorder, AUD, alcohol use disorder, DUD, drug use disorder, ADHD, attention deficit-hyperactivity disorder, ODD, oppositional defiant disorder, GAD, generalized anxiety disorder, OCD, obsessive-compulsive disorder, PTSD, posttraumatic stress disorder, cPTSD, complex posttraumatic stress disorder, DSM, Diagnostic and Statistical Manual of Mental Disorders, HC, healthy controls, CC, clinical controls, PFC, pre-frontal cortex, OFC, orbito-frontal cortex, ACC, anterior cingulate cortex, DLPFC, dorsolateral pre-frontal cortex, BA24, Brodmann area, ILF, inferior longitudinal fasciculus.

**Table 5:** Semi-structured interviews.

Name (Abbreviation)	Personality Disorders Assessed	Characteristics	Validated for Adolescents
Structured Clinical Interview for DSM-IV Axis II Disorders (SCID-II)	All personality disorders	Items grouped by personality-disorder type	Yes
Diagnostic interview for DSM-IV Personality Disorders (DIPD-IV)	All personality disorders	Items grouped by personality-disorder type	
International Personality Disorders Examination (IPDE)	All personality disorders in DSM-IV and ICD-10	Items grouped by topic (i.e., work, self, interpersonal, affect, reality testing, impulse control)	
Structured Interview for DSM-IV Personality Disorders (SIDP-IV)	All personality disorders	Items grouped by personality-disorder type or by topic	
Structured Clinical Interview for the DSM-5 Alternative Model for Personality Disorders Module III (SCID-5-AMPD)	BPD and five other personality disorders	Items grouped by personality-disorder type, based on DSM-5 AMPD	
Revised Diagnostic Interview for Borderlines (DIR-B)	BPD only	Items grouped by area of functioning (impulsivity, affect, cognition, interpersonal relationships)	
Childhood Interview for DSM-IV Borderline Personality Disorder (CI-BPD)	BPD only	Specifically designed for adolescents	Yes
Borderline Personality Disorder Severity Index-IV (BPDSI-IV)	BPD only	Dimensional, short-interval change measure, with adolescent and parent versions	Yes
Zanarini Rating Scale for Borderline Personality Disorder (ZAN-BPD)	BPD only	Dimensional, short-interval change measures	
Alcohol Use Disorder and Associated Disabilities Interview Schedule-5 (AUDADIS-5)	BPD, antisocial personality disorder (ASPD) and schizotypal personality disorder (STPD)	Structured interview for lay-person administration used in the NESARC (National Epidemiologic Survey on Alcohol and Related Conditions)	

BPD, borderline personality disorder; ASPD, antisocial personality disorder; DSM, Diagnostic and Statistical Manual of Mental Disorders; ICD-10, International Classification of Diseases.

**Table 6:** Self-report questionnaires.

Name (Abbreviation)	Personality Disorders Dssessed	Aim	Characteristics	Validated for Adolescents
Personality Diagnostic Questionnaire-4 (PDQ-4)	All personality disorders	Diagnosis	Includes questions on clinical significance	
Personality Assessment Inventory (PAI)	BPD and ASPD	Diagnosis	Assesses identity problems, negative relationships, affective instability and self-harm; has an adolescent version	Yes
Borderline Symptom List (BSL)	BPD	Diagnosis	Full and short version	
Five-Factor Borderline Inventory (FFBI)	BPD	Diagnosis	Based on the Five-Factor Model of personality traits	
Schedule for Nonadaptive and Adaptive Personality-II (SNAP-II)	All personality disorders and traits	Pathological personality traits assessment; can be scored for diagnosis	Distinguishes higher-order factors and lower-order traits	Yes
Dimensional Assessment of Personality Pathology-Basic Questionnaire (DAPP-BQ)	All personality disorders and traits	Pathological personality traits assessment	Assesses identity problems, insecure attachment, affective lability and self-harm	
Minnesota Multiphasic Personality Inventory-2-Restructured Form (MMPI-2-RF)	All personality disorders and traits	Pathological personality traits assessment	Dimensional	
Personality Inventory for DSM-5 (PID-5)	All personality disorders and traits	Pathological personality traits assessment	Based on the DSM-5 Alternative Model for Personality Disorders (AMPD)	
McLean Screening Instrument for BPD (MSI-BPD)	BPD	Screening	Ten items; translated into multiple languages; used in adults and adolescents	Yes
Borderline Personality Questionnaire (BPQ)	BPD	Screening	Used in adults and adolescents	Yes
Borderline Personality Features Scale for Children (BPFSC)	BPD	Screening	Dimensional scale designed to assess children and adolescents; has child and parent version	Yes
Severity Indices of Personality Problems (SIPP-118)	-	Impairment in personality functioning assessment	Includes five domains of personality functioning	
General Assessment of Personality Disorder (GAPD)	-	Impairment in personality functioning assessment	Assesses self or identity problems and interpersonal dysfunction	
Level of Personality Functioning Scale Self-Report (LPFS-SR)	-	Impairment in personality functioning assessment	Based on the DSM-5 AMPD	

BPD, borderline personality disorder; ASPD, antisocial personality disorder; DSM, Diagnostic and Statistical Manual of Mental Disorders; ICD-10, International Classification of Diseases.

## Data Availability

Not applicable.
